# Personal

**Published:** 1907-04

**Authors:** 


### PERSONAL.

Dr. J. H. White, of the United States Public Health and Marine Hospital Service, who directed the campaign which stamped out yellow fever in New Orleans in 1905, has received an appointment as supervising inspector of maritime quarantine in Louisiana, Mississippi and the Central American fruit ports from Surgeon General Wyman, of the Marine Hospital Service. Dr. White's appointment is a step in establishing the national quarantine in Louisiana, supplanting the state system.

Dr. Carlos E. Bowman, of Alden, Erie County, N. Y., was recently elected president of that village. He has practised his profession there for over twenty years and is spoken of as one of the most respected citizens of Alden. He is and has been for a number of years health officer of the village and takes an active interest in public affairs.

Dr. I. A. M. Dike, of York, Livingston County, N. Y., was re-elected supervisor at the recent spring election. Dr. Dike has practised his profession in that town for more than twenty-five years and is one of the prominent citizens of that community.

Dr. Vertner Kenerson, of Buffalo, captain and assistant surgeon in the 74th regiment of infantry, N.Y.N.G., will resign his commission, it is announced, at an early day. His successor has not been determined upon.

Dr. Prescott Le Breton, of Buffalo, May 1, 1907, will remove from 20 Carlton Street to 23 Irving Place, where he will establish his residence and offices. Hours: 9 to 11 and 1 to 2.

